# Analysis and findings from the Zimbabwe supply chain human resource assessment

**DOI:** 10.1186/2052-3211-7-S1-P1

**Published:** 2014-12-17

**Authors:** Brian Serumaga, Rachel Kearl, Misheck Ndlovu, Tinei Chisike

**Affiliations:** 1 USAID | DELIVER PROJECT, John Snow, Inc. Washington DC, USA; 2 Ministry of Health and Child Welfare, Harare, Zimbabwe; 3 USAID | DELIVER PROJECT Zimbabwe, John Snow Inc., Harare, Zimbabwe

## Background

Although progress has been made in growing Zimbabwe’s public sector health commodity supply chain, human resource challenges remain. To understand and address these challenges, the Ministry of Health and Child Welfare (MOHCW) Directorate of Pharmacy Services (DPS), with support from the USAID|DELIVER Project, conducted a human resources (HR) capacity assessment in March 2012 that: documented the state of supply chain HR capacity, identified opportunities to build HR capacity, documented professionalization efforts of supply chain personnel.

## Method

We used the USAID|DELIVER Project Human Resource Capacity Development Assessment Guide and Tool to evaluate Zimbabwe’s public health supply chain HR based on five components: powerful constituencies, policies and plans, workforce development, workforce performance management, and professionalization.

Investigators: gathered data using focus groups at central, provincial, district, and health facility levels, surveyed two urban (Harare, Bulawayo) and two rural (Matebeleland South, Mashonaland East) provinces, and surveyed MOHCW, city, and mission-managed facilities.

## Results

The assessment team carried out a detailed analysis of each component based on collected data. They examined internal strengths and weaknesses of the system and external opportunities and threats. Broad findings included: limited funding for positions with supply chain responsibilities and significant reliance on donors for the staffing of key positions, inadequate coordination and communication of workforce resources and expectations among MOHCW’s departments (pharmacy services, nursing services, and human resources), and low staff retention due mainly to lower compensation rates for staff in public health facilities compared to colleagues in the private sector.

## Discussion

The following support would strengthen HR management for the health supply chain in Zimbabwe: create an HR coordination group that includes senior management from different health departments in the MOHCW, incorporate supply chain cadres into the existing HR retention scheme supported by The Global Fund To Fight AIDS, Tuberculosis and Malaria (GFATM), utilize district medical officers to improve HR information dissemination for supply chain cadres across all levels, update the staffing structure and clarify roles and expectations to reflect current supply chain requirements, implement an enhanced mentoring program to accelerate development and retention of supply chain cadres, and develop and implement supply chain pre-service training for allied health cadres.

## Lessons learned

This assessment found challenges for health workers with supply chain responsibilities in Zimbabwe. Nevertheless, the assessment also revealed that practical and less costly interventions could yield substantial improvements in the short and long terms. These interventions include better coordination, improved commissioning of existing resources, and long-term investments in training.

